# Use of pirfenidone in fibrotic interstitial lung diseases and beyond: a review

**DOI:** 10.3389/fmed.2024.1411279

**Published:** 2024-08-06

**Authors:** Mingfeng Han, Qijia Liu, Zhe Ji, Lili Jin, Wenyu Jin, Zhonggao Gao

**Affiliations:** ^1^School of Pharmacy, Yanbian University, Yanji, Jilin, China; ^2^Ruibo International Business School, Beijing, China; ^3^School of Finance, Renmin University of China, Beijing, China; ^4^Department of Dermatology, Yanbian University Hospital, Yanji, Jilin, China; ^5^State Key Laboratory of Bioactive Substance and Function of Natural Medicines, Department of Pharmaceutics, Institute of Materia Medica, Chinese Academy of Medical Sciences and Peking Union Medical College, Beijing, China

**Keywords:** fibrotic interstitial lung diseases, pirfenidone, CTD-ILD, Post-COVID-19 pulmonary fibrosis, adverse events

## Abstract

The pathophysiological mechanisms involved in fibrotic interstitial lung diseases (FILDs) are akin to those observed in idiopathic pulmonary fibrosis (IPF), implying the potential for shared therapeutic approaches. Pirfenidone exhibits antifibrotic and anti-inflammatory properties, making it the first small-molecule drug approved for treating IPF. Pirfenidone has been utilized in IPF treatment for more than one decade. However, guidelines for progressive pulmonary fibrosis (PPF) treatment suggest that further research and evidence are needed to fully comprehend its efficacy and safety across various PPF subtypes. In recent years, numerous studies have explored the use of pirfenidone in treating non-IPF FILD. Herein, we provide an overview of the latest research data on application of pirfenidone in occupational-related ILD, connective tissue disease-associated ILD, post-coronavirus disease-2019 pulmonary fibrosis, and other conditions. We summarize the level of evidence and highlight challenges associated with using pirfenidone in different FILDs to offer clinical guidance.

## 1 Introduction

Fibrotic interstitial lung diseases (FILDs) are the most common manifestation of diffuse parenchymal interstitial lung diseases (ILDs) (1). FILDs have a wide range of pathological features, clinical manifestations, imaging findings, and outcomes (2).

Idiopathic pulmonary fibrosis (IPF) is the most archetypal FILD. IPF is characterized by self-sustaining and progressive fibrosis, deteriorating lung function (e.g., forced vital capacity (FVC), carbon monoxide diffusing capacity (DLco), worsening symptoms, declining exercise capacity, increased risk of hospitalization, and early death (3). The median interval between the diagnosis and death for patients suffering from IPF is 3–5 years (4).

Since proposal of the concepts of PF-ILD and progressive pulmonary fibrosis (PPF) (5–7), FILDs have gained increasing attention, including connective tissue disease-interstitial lung disease (CTD-ILD) (8), environmental exposure-based ILD (9), and post-coronavirus disease-2019 (COVID-19)-related PF (10). These disease processes are similar to IPF, but standard treatment is lacking (11). Whether non-IPF-based FILDs can be treated with two approved antifibrotic drugs, pirfenidone or nintedanib, is not known.

Pirfenidone (5-methyl-1-phenyl-2(1H)-pyridone; Chemical Abstracts Service number: 53179-13-8) is an antifibrotic agent given via the oral route. It has been used widely in the clinical treatment of IPF since it was approved in Europe and China in 2011 and the USA in 2014. The efficacy of pirfenidone against IPF has been demonstrated in several randomized controlled placebo trials and over one decade of real-world experiences. Pirfenidone can mitigate the decline in lung function, reduce the risk of death, and lengthen progression-free survival (12, 13).

In recent years, there has been an increasing focus on the use of pirfenidone for non-IPF FILDs, particularly since introduction of the concept of PF-ILD and PPF. The American Thoracic Society (ATS), European Respiratory Society (ERS), Japanese Respiratory Society (JRS) and Asociación Latinoamericana de Tórax (ALAT) PPF Clinical Practice Guidelines Committee has recommended the use of antifibrotic agents for patients with PPF who do not respond to standard therapy for a FILD (5). This recommendation highlights the importance of exploring alternative treatment options for individuals facing challenges in managing their condition. Specifically, pirfenidone has shown potential for managing PPF in patients not suffering from IPF. The ATS/ERS/JRS/ALAT PPF Clinical Practice Guidelines Committee conducted a comprehensive review of two trials on pirfenidone in populations that met the PPF definition: RELIEF (14) and U-ILD (15). Findings from both trials demonstrated that pirfenidone mitigated the rate of decline in lung function among patients with PPF and exhibited a favorable safety profile. However, the level of evidence supporting these conclusions was limited. Twenty-one out of 34 experts on the ATS/ERS/JRS/ALAT PPF Clinical Practice Guidelines Committee, provided a “conditional recommendation” for pirfenidone. This recommendation indicates a consensus among these experts regarding its potential benefits, but further research and evidence are needed to fully understand its efficacy and safety across various subtypes of PPF.

**FIGURE 1:**
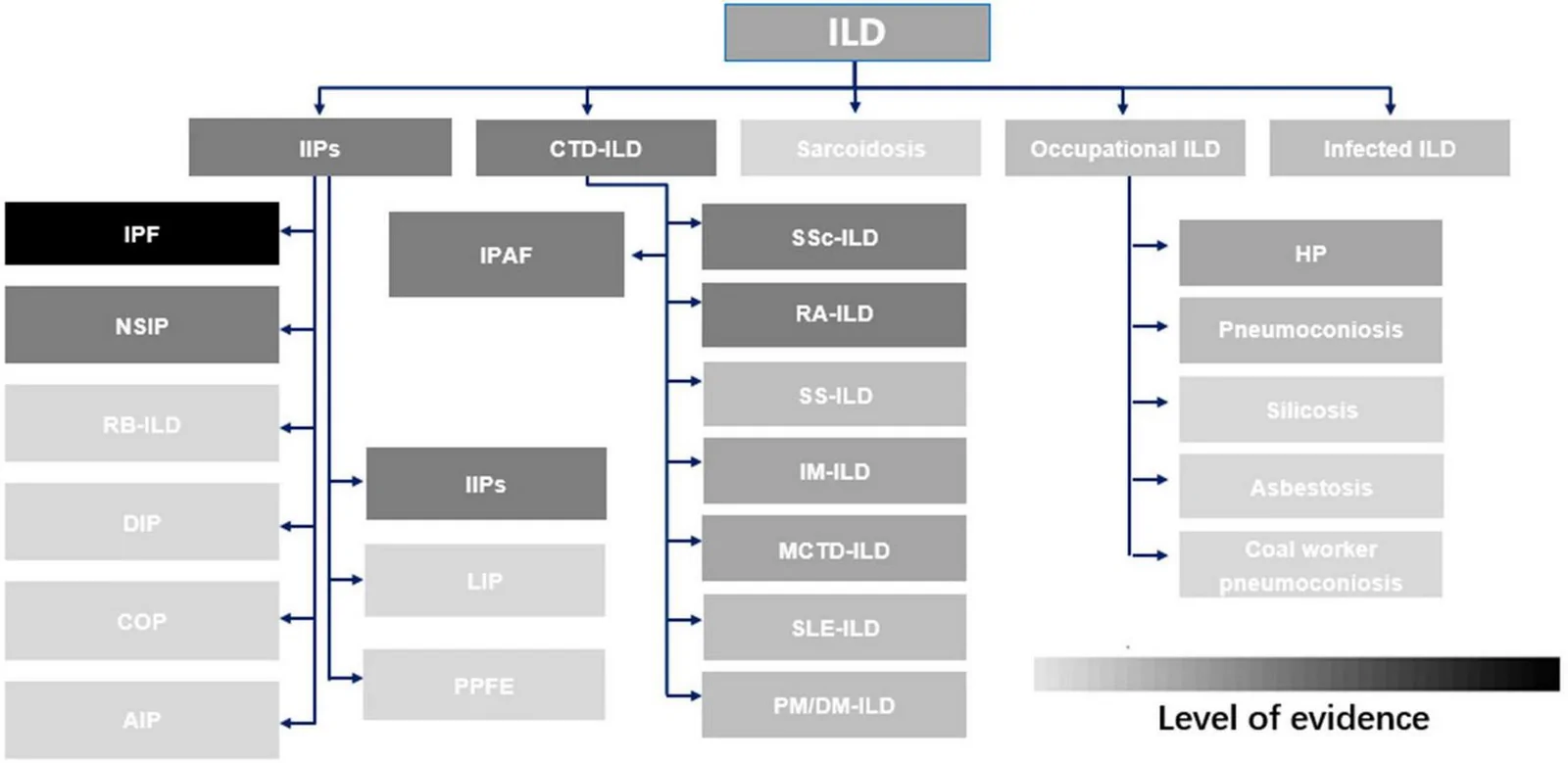
Evidence supporting the use of pirfenidone in common interstitial lung diseases. ILD, interstitial lung disease; IIP, idiopathic interstitial pneumonia; CTD-ILD, connective tissue disease-associated interstitial lung disease; IPF, idiopathic pulmonary fibrosis; NSIP, non-specific interstitial pneumonitis; RB-ILD, respiratory bronchiolitis interstitial lung disease; DIP, desquamative interstitial pneumonia; COP, cryptogenic organizing pneumonia; AIP, acute interstitial pneumonia; IPAF, Interstitial pneumonia with autoimmune features; LIP, lymphocytic interstitial pneumonia; PPFE, pleuropulmonary fibroelastosis; IIP, idiopathic interstitial pneumonia; SSc-ILD, systemic sclerosis-associated interstitial lung disease; RA-ILD, rheumatoid arthritis-associated interstitial lung disease; SS-ILD, Sjögren’s syndrome-related interstitial lung disease; IM-ILD, inflammatory myositis-associated interstitial lung disease; MCD-ILD, mixed connective tissue disease-associated interstitial lung disease; SLE-ILD, systemic lupus erythematosus-associated interstitial lung disease; PM/DM-ILD, polymyositis/dermatomyositis-associated interstitial lung disease; HP, hypersensitivity pneumonitis.

The efficacy of pirfenidone has been investigated extensively in various ILDs through numerous clinical studies and basic-science experiments, but the level of evidence is heterogeneous (Figure 1). A comprehensive systematic review summarizing the data on the effectiveness in managing IPF and non-IPF FILDs is lacking.

We conducted a search across databases (Medline, EMBASE, and Cochrane) for relevant studies published within the previous 5 years to summarize the research progress made with pirfenidone in treating various types of FILDs. We also highlighted limitations in understanding and proposed future research directions.

## 2 Investigation of IPF in off-label settings

The effectiveness of pirfenidone in delaying the decline in lung function and improving progression-free survival has been demonstrated through multiple phase-3 randomized, controlled placebo trials in patients with IPF. The results of these trials have resulted in regulatory approval of pirfenidone for IPF treatment in various countries. Over the last decade, the utilization of pirfenidone in treating IPF has generated substantial real-world data from numerous nations (16–23).

In many countries, use of pirfenidone is limited to patients with mild-to-moderate IPF, excluding those with advanced IPF. A *post hoc* analysis of six clinical studies of pirfenidone [ASCEND (12), CAPACITY 004 and 006 (13), RECAP (24), PASSPOR, SP-IPF (25)] compared the efficacy and safety of pirfenidone in advanced IPF vs. non-advanced IPF. The analysis highlighted the benefit of pirfenidone treatment in patients with advanced IPF in terms of FVC decline from baseline and prevalence of all-cause mortality. New safety signals for pirfenidone in patients with advanced IPF were not identified. Data from a phase-2b trial showed that pirfenidone plus placebo given for up to 52 weeks in patients with advanced IPF and a risk of pulmonary hypertension was well tolerated. In that trial, pirfenidone plus placebo was used as a control group (26). Pirfenidone and nintedanib were approved by the US Food and Drug Administration for the treatment of patients with IPF regardless of disease severity. Evidence of new safety concerns relating to the use of pirfenidone in patients with advanced IPF has not emerged in over 10 years of real-world experience in the USA (27). The countries and regions that approve pirfenidone only for mild-to-moderate IPF should, therefore, consider the potential benefits of extending its use against advanced IPF.

The disease course of IPF is complex and often accompanied by comorbidities (28). In certain individuals diagnosed with IPF, concurrent lung cancer can be observed, which necessitates surgical intervention (29). However, these patients frequently experience acute exacerbations following lung resection. Postoperative acute exacerbation is the primary cause of death after surgery for lung cancer, with mortality ranging from 33.3 to 100% being reported (30). The predicted prevalence of acute exacerbations within postoperative day (POD)30 for a cohort of patients was 10.7% based on the risk scoring system of the Japanese Association for Chest Surgery, whereas that for pirfenidone treatment was 3.6%, indicating that pirfenidone reduced the occurrence of acute exacerbations significantly (31). Another study found a significant association between perioperative pirfenidone treatment and incidence of postoperative AE within POD30 (*p* = 0.045) and POD90 (*p* = 0.04) (31). The PEOPLE study was a multicenter prospective phase-2 trial. It showed that only 5.1% (2/39) of the full analysis set and 2.8% (1/36) of the per-protocol set suffered postoperative acute exacerbations for IPF in patients suffering from lung cancer who received pirfenidone (1,200 mg daily) for ≥ 2 weeks before surgery. A grade-5 adverse event (death) occurred in one patient after an acute exacerbation of IPF, but other adverse events of grade 3–5 were not observed. Perioperative pirfenidone treatment for IPF in patients with lung cancer is safe, and promising for reducing acute exacerbations of IPF after lung-cancer surgery (32). In the PEOPLE study, spirometry data were missing from some post-baseline visits because of site restrictions and early terminations, and the trial was not randomized but instead a single-arm study in which historical data served as the control. To confirm the efficacy of perioperative pirfenidone treatment for postoperative acute exacerbations, the PIII-PEOPLE study (NEJ034), which is a phase-3, multicenter, prospective, randomized controlled clinical trial, is ongoing (33).

Patients with IPF experience a decline in lung function throughout all stages of disease progression. Use of antifibrotic drugs can provide benefits at any stage, not just for those with mild-to-moderate progression. Also, the perioperative administration of pirfenidone may provide benefits for patients suffering from IPF.

## 3 ILD due to occupational and environmental exposures

Occupational and environmental exposures are significant contributors to FILD development. Progression in patients with a FILD closely resembles that observed in people with IPF (9, 34). The potential of pirfenidone for treating ILDs caused by environmental exposures is substantial.

Fibrotic hypersensitivity pneumonitis (FHP) is the most common FILD associated with environmental and occupational exposures. FHP leads to progressive disease progression based on symptomatic, functional, and radiographic features (35, 36). The RELIEF trial demonstrated the promising efficacy of pirfenidone in treating progressive fibrosing interstitial lung disease (PF-ILD). The most frequent diagnosis of ILD among 127 patients was chronic hypersensitivity pneumonitis (57 patients, 45%), and an additional six (5%) patients with asbestos-induced lung fibrosis were included. Patients with occupational and environmental exposures accounted for half of the enrolled patients in the RELIEF trial. The findings of the RELIEF trial can be applied to patients with environmental exposure-based ILDs, particularly in those with hypersensitivity pneumonitis [albeit to a certain extent (14)]. The first double-blinded, randomized, placebo-controlled trial conducted exclusively in patients with FHP using pirfenidone was terminated prematurely due to the COVID-19 pandemic. In addition, the sample size was inadequate for detecting a significant effect of pirfenidone therapy on the primary endpoint. However, consistent positive trends in FVC% were observed in the pirfenidone group compared with that in the placebo group. Furthermore, pirfenidone demonstrated an acceptable safety profile and improved PFS in patients with FHP (37). The findings of this phase-2 trial are consistent with those from an open-label trial conducted earlier in 2024, which investigated the efficacy of pirfenidone in combination with prednisone and azathioprine among 22 patients diagnosed with hypersensitivity pneumonitis. After 1 year of treatment, significant improvement in FVC was not observed among patients receiving pirfenidone, but they experienced an improved quality of life while pirfenidone maintained an acceptable safety profile (38). Additional large-scale trials are required for approval of pirfenidone as a treatment option for FHP.

Silicosis is an occupational disease resulting from prolonged inhalation of silica particles. It is characterized by pulmonary dysfunction, persistent inflammation in the lungs, and irreversible fibrosis (39). Despite global efforts to minimize workers’ exposure to silica particulate matter, a significant number of new silicosis cases continues to be reported (particularly in developing nations). In recent years, limited advancements have been made regarding the development of therapeutic drugs for silicosis. In an animal study, pirfenidone demonstrated efficacy in improving various stages of silicosis (40). Pirfenidone could inhibit interleukin (IL)-17A production, which plays a crucial part in the pathogenesis of silicosis. However, the progression of occupational diseases is slow, with a long period of clinical observation and high specificity. Large randomized controlled studies on antifibrotic drugs for treating occupational diseases have not been carried out. Given the challenges in conducting such trials for conditions such as silicosis, long-term retrospective studies are recommended to investigate if pirfenidone can benefit patients with occupational diseases.

## 4 CTD-ILD

CTDs are autoimmune disorders characterized by chronic non-infectious inflammation affecting blood vessels and connective tissue throughout the body. They can involve all tissues and organs, with the lungs being commonly affected. ILD, pulmonary hypertension, and pleurisy can manifest in CTDs and CTD-like diseases, with ILD being the most prevalent condition (41, 42). The prevalence of ILD varies significantly (3 to 70%) due to the different methods used to detect it. Furthermore, ILD associated with different CTDs exhibits distinct clinical manifestations, imaging findings, and pathological features that contribute to diverse disease progression and outcomes (43). CTD-ILD management should adhere to the “double target” principle, which entails controlling the underlying CTD and ILD progression. Different subspecies of CTD-ILD require distinct approaches for effective management (44).

Rheumatoid arthritis (RA) shares certain mechanisms and functions with CTD. ILD is found commonly in patients with RA (RA-ILD), affecting up to 60% of individuals with RA and leading to premature death in 10% (9). The TRAIL1 trial was the inaugural multicenter, randomized, double-blind, placebo-controlled phase-2 study investigating the safety, tolerability, and efficacy of antifibrotic agents in patients suffering from RA-ILD (45). The pirfenidone group exhibited a slower annual decline in FVC compared with that in the placebo group, with values of 66 mL and 146 mL, respectively. This difference was particularly pronounced in patients with pattern of usual interstitial pneumonia, for which the decline in FVC was 43 mL for the pirfenidone group compared with 169 mL for the placebo group.

Systemic sclerosis (SSc) is a rare and heterogeneous systemic autoimmune disease characterized by excessive production of collagen as well as fibrosis in the skin and internal organs (46). ILD is a common complication of SSc, with high-resolution computed tomography revealing ILD in 40–75% of patients. Furthermore, ILD serves as the primary cause of death among individuals with SSc (47). To investigate the safety and tolerability of pirfenidone for treating patients with systemic sclerosis-associated interstitial lung disease (SSc-ILD), an international, multicenter, randomized, open-label phase-2 study (LOTUSS) was conducted (48). Sixty-three patients were enrolled and 96.8% experienced at least one treatment-emergent adverse event (TEAE). Nausea, headache, and fatigue were the most frequently reported TEAEs. The frequency and type of TEAEs were comparable between 2-week and 4-week dose-adjustment groups. Most TEAEs were mild or moderate in severity once pirfenidone reached its full dosage level; no life-threatening TEAEs or deaths occurred within 3 weeks. The TEAEs observed in that study were consistent with the findings from three randomized placebo-controlled phase-3 trials of pirfenidone in IPF (*n* = 1247) and the long-term safety evaluation of pirfenidone in IPF (*n* = 789). Those trials demonstrated that the safety and tolerability of pirfenidone in patients with SSc-ILD was comparable with that observed in patients with IPF. The LOTUSS trial provided stable results regarding disease outcomes in patients with SSc-ILD, but the study duration was insufficient to assess efficacy. Future trials investigating the efficacy of pirfenidone in patients with SSc-ILD should consider expanding the study population and extending the duration of observation.

The Sjögren’s syndrome (pSS) is an autoimmune disorder primarily affecting the salivary and lacrimal glands (49). In certain cases, patients with pSS may experience lung injury, including the development of interstitial pneumonia (50). Consensus regarding the treatment approach for interstitial pneumonia secondary to pSS is lacking. A randomized controlled trial was conducted to investigate the potential positive clinical implications of pirfenidone in patients with pSS (51). The study cohort comprised 120 participants divided into two groups: a control group receiving hydroxychloroquine and prednisone, and an observation group receiving pirfenidone in addition to standard treatment. A superior improvement in lung function within the observation group compared with that in the control group was observed (*p* < 0.05). Following treatment, both groups exhibited significant enhancements in the Warrick score, but the observation group displayed a more pronounced decrease than that of the control group (*p* < 0.05). Moreover, the post-treatment Warrick score improved significantly in both groups compared with the pre-treatment score, with the observation group achieving a significantly higher Warrick score than the control group (*p* < 0.05). The potential of pirfenidone to enhance pulmonary function and alleviate cough symptoms in patients with pSS-associated non-specific interstitial pneumonia may contribute to an improved quality of life for these individuals.

The diagnosis of patients with ILD involves the underlying characteristics of autoimmune disease. These features include (but are not limited to) joint swelling, skin dysfunction, and organ dysfunction. Many patients show the characteristics of potential autoimmune diseases, but they do not meet the criteria for a diagnosis of a specific CTD. This conundrum poses a challenge for clinicians in the accurate diagnosis and management of the condition. To address this issue, a new diagnostic category called “interstitial pneumonia with autoimmune features” (IPAF) has been proposed (52). IPAF allows for the identification and classification of individuals who demonstrate clinical features suggestive of an underlying autoimmune process but fall short of meeting established criteria for a CTD (53). A retrospective study of 242 patients with IPAF revealed a significant increase in FVC% (10.44%) after 12 months of pirfenidone treatment, whereas the control group experienced a slight decrease (1.18%) (*p* = 0.013). Also, patients receiving pirfenidone required a lower dose of glucocorticoids compared with the control group. That study revealed that the adverse effects observed in patients with IPAF treated with pirfenidone were comparable with those experienced by patients with IPF and were, in general, considered acceptable (54).

Ongoing clinical trials are investigating the long-term effects and optimal dosing strategies for pirfenidone in patients with CTD-ILD. Such studies aim to provide further evidence regarding its safety profile and efficacy in different subtypes of CTD-ILD.

Overall, a comprehensive therapeutic strategy for CTD-ILD should encompass the management of the underlying CTD and pulmonary involvement. Additional research is needed to fully understand the role of pirfenidone in treating patients with CTD-ILD and progressive fibrosis. However, evidence suggests that it may offer a valuable therapeutic option for patients with UIP or those experiencing worsening lung scarring due to their CTD.

## 5 Post-COVID-19 pulmonary fibrosis

Severe acute respiratory syndrome 2 (SARS-CoV-2) infection can induce acute lung injury and, in some cases, lead to the development of acute respiratory distress syndrome, which is typically accompanied by a decline in lung function similar to that observed in pulmonary fibrosis (10, 55). Certain patients (particularly hospitalized cases) may experience excessive tissue repair resulting in fibrosis known as “post-COVID-19 pulmonary fibrosis,” which necessitates urgent attention and appropriate management (56, 57).

The precise mechanism underlying post-COVID-19 pulmonary fibrosis is associated with the activation of transforming growth factor (TGF)-β1, which regulates the release of extracellular proteins, fibroblast activity, fibroblast migration, and myofibroblast conversion. Pirfenidone inhibits the accumulation and recruitment of inflammatory cells, proliferation of fibroblasts, and deposition of the extracellular matrix in response to TGF-β1 and other proinflammatory cytokines (58, 59). Pirfenidone downregulates expression of TGF-β1 (protein effector involved in SARS-CoV-2 entry and TGF-β1 activation), thereby reducing the pathogenesis of SARS-CoV-2 (60). Moreover, pirfenidone modulates signaling pathways such as Wingless/Int, Yes-associated protein/transcription co-activator PDZ binding motif, and Hippo signaling pathways that contribute to the pathogenesis of post-COVID-19 pulmonary fibrosis (61). Hence, the anti-inflammatory and antifibrotic properties of pirfenidone may mitigate post-COVID-19 pulmonary fibrosis.

A study conducted during the early stages of the COVID-19 outbreak (31 January to 3 March 2020) investigated the efficacy of pirfenidone treatment in patients with severe COVID-19 with blood oxygen saturation < 94% and a low ratio of arterial partial pressure of oxygen to fraction of inhaled oxygen (62). Compared with the non-pirfenidone group receiving standard treatment, patients treated with pirfenidone (400 mg, t.i.d.) exhibited higher levels of IL-2R (*p* = 0.010) and TNF-α (*p* = 0.010), as well as improved fibrosis-related scores on imaging tests, such as consolidation (*p* = 0.007), Ground-Glass Opacity (GGO) and reticular formation. Furthermore, significant differences were not observed between the pirfenidone group and control group with regard to adverse effects in this preliminary study, suggesting that it could be safe for subsequent trials involving patients with COVID-19. A randomized controlled study assigned 100 adult patients suffering from COVID-19 and the “cytokine storm” and admitted to the intensive care isolation unit into two groups: pirfenidone added to standard therapy, or standard protocol only. The prevalence of pulmonary fibrosis during the cytokine storm did not differ significantly between the pirfenidone group and standard group (29.8% vs. 35.8%). However, a significantly higher proportion of patients in the pirfenidone group were discharged from hospital without progression of pulmonary fibrosis (21.3% vs. 5.7%, *p* = 0.006) (63). An open-label pilot trial involving 60 patients with COVID-19 was undertaken. Seventeen patients received pirfenidone and 19 received corticosteroids. Study parameters were evaluated at baseline and after 6 weeks. The antifibrotic effects of pirfenidone were found to be superior to those of corticosteroids (*p* < 0.001). These observations suggested that early treatment with pirfenidone in patients with severe COVID-19 may reduce the risk of developing post-COVID-19 pulmonary fibrosis (61). A single-center retrospective study involved assessment of patients with post-COVID-19 pulmonary fibrosis treated with pirfenidone (2,400 mg/day) for 12–24 weeks showed that pirfenidone reduced the risk of development of pulmonary fibrosis (64).

However, caution should be exercised when employing antifibrotic therapy in patients with post-COVID-19 pulmonary fibrosis due to the limited number of randomized controlled trials assessing the efficacy and safety of pirfenidone. Additional clinical data are required to ascertain the specific effects of pirfenidone in this population. Also, the clinical impact of antifibrotic therapies on post-COVID-19 pulmonary fibrosis must be determined considering that various types of viral and bacterial infections can also lead to pulmonary fibrosis with similar mechanisms and progression as observed in patients with post-COVID-19 pulmonary fibrosis. This knowledge will inform management strategies during a future pandemic. Furthermore, considering the distinct course of pulmonary fibrosis induced by COVID-19 and other infections compared with pulmonary fibrosis, one must consider three aspects when assessing the efficacy of antifibrotic therapy for post-COVID-19 pulmonary fibrosis. First, one must ascertain if there are diverse response patterns among patients at different stages of disease or with different subtypes of disease, and identify factors that may influence therapeutic outcomes. Second, empirical studies are warranted to determine the optimal dose and duration of treatment. Third, comprehensive surveillance and management protocols should be implemented to monitor complications, drug–drug interactions, and concerns regarding long-term safety.

## 6 Adverse events

At the recommended dosing, the oral antifibrotic medications approved to treat IPF (nintedanib or pirfenidone) can be associated with increases in levels of liver enzymes and gastrointestinal side-effects (65–68). In general, pirfenidone is well tolerated, with the most common TEAEs observed in clinical trials were related to the gastrointestinal system and skin. These adverse events are, in general, mild-to-moderate in severity and rarely result in treatment discontinuation (69).

The ASCEND and CAPACITY trials were conducted to evaluate the efficacy and safety of pirfenidone in patients with IPF. A significant difference in the major adverse effects between the two trials was not observed. These findings were consistent with other real-world data collected from phase-3 studies. It is worth noting that the criteria for adverse effects varied among the studies, therefore caution should be exercised when interpreting the conclusion of no difference in adverse effect occurrence. Interestingly, significant differences were observed among the common adverse effects examined in both the PPF RCT trials and the IPF trials (Table 1). For instance, Rash showed a statistically significant difference (*p* = 0.002, 95% CI: 0.250–0.755), as well as Dizziness (*p* = 0.001, 95% CI: 0.217–0.547). These discrepancies may be attributed to the majority of adverse reactions being classified as grade I or grade II, resulting in considerable variability. Besides, several factors may have contributed to discrepancies: differences in study duration, as well as potentially greater disease severity and drug sensitivity among patients with IPF. In addition, certain patient populations may respond differently to treatment due to genetic or environmental factors.

**TABLE 1:** Common adverse effects of pirfenidone.

Adverse reaction	IPF			PPF			*p*
	CAPACITY trial (*n* = 345)	ASCEND trial (*n* = 278)	Total	U-ILD trial (*n* = 120)	RELIEF trial (*n* = 64)	Total	
Gastrointestinal disorder	191 (55%)	141 (50.7%)	53%	60 (47%)	33 (52%)	51%	0.896
Rash	111 (32%)	78 (28.1%)	30%	13 (10%)	7(11%)	13%	0.001
Dizziness	63 (18%)	49 (17.6%)	18%	10 (8%)	8 (13%)	10%	0.002
Photosensitivity reaction	42 (12%)	36 (13%)	13%	10 (8%)	5 (8%)	8%	0.103

The Pearson chi-square test was employed due to the minimum expected value of each data set being greater than 5 and the sample size exceeding 40.

Adverse events caused by antifibrotic drugs can lead to treatment interruptions and impact disease progression. The ASCEND and CAPACITY trials reported a prevalence of discontinuation of 14.4% and 15% in patients with IPF, respectively, whereas the RELIEF trial demonstrated a prevalence of discontinuation of 15% in patients with PF-ILD compared with those suffering from IPF. In a real-world setting, data from China revealed that the prevalence of discontinuation of pirfenidone after 6 months was 18.37%, slightly surpassing the prevalence observed in randomized controlled trials due to financial constraints leading patients to discontinue treatment. However, upon exclusion of 2.93% of discontinuations attributed solely to financial reasons, the real-world prevalence of discontinuation aligned with that reported in randomized controlled trials. Notably, the prevalence of discontinuation at 1 year for pirfenidone was 24.39%, whereas the prevalence of long-term discontinuation reached 32.68% (70). Occasionally, reducing the dose can manage adverse events effectively. Data from Korea indicate that lowering the daily dose of pirfenidone from 1,800 mg to 1,200 mg can delay the decline of FVC and DLco significantly in patients (71), suggesting that dose reduction could maintain efficacy while minimizing the risks of adverse events.

Aerosol administration improves the efficacy and safety of many drugs by increasing delivery to lung tissue and decreasing systemic exposure (72). A randomized, double-blinded, placebo-controlled, dose-escalation phase-1 trial investigated the effect of aerosolized pirfenidone (AP01) delivered *via* the eFlow^®^ nebulizer (PARI, Starnberg, Germany) in healthy volunteers and patients with IPF. The eFlow nebulizer delivered > 40% of the dose to the lung and enabled alveolar delivery. AP01 was well tolerated by healthy volunteers and patients. The highest dose of AP01 tested (100 mg) achieved a 35-fold higher peak concentration in the epithelial lining fluid with only about 1/15 systemic exposure compared with the approved dose of pirfenidone (801 mg, t.i.d., p.o.) (73). A phase-1b, randomized, open-label, dose–response trial in patients with IPF of AP01 showed that the side-effects commonly associated with oral pirfenidone in other clinical trials were less frequent with inhaled pirfenidone. The mean FVC% predicted for patients with IPF remained stable in the group taking pirfenidone at 100 mg two-times daily (74). Further studies of inhaled pirfenidone and other new formulations which could reduce the number and severity of side effects are warranted.

Despite some limitations, overall the evidence suggests that pirfenidone is a safe and efficacious treatment option. Implementation of dose adjustments and introduction of novel delivery modalities may help to mitigate adverse effects. The observed adverse events in new FILDs were consistent with those reported in IPF studies. The management of adverse events in FILDs can be derived from the disease-management strategies employed in IPF. New formulations of pirfenidone also hold promise in terms of reducing adverse reactions and enhancing medication compliance.

## 7 Discussion

The antifibrotic drug pirfenidone underwent phase-3 trials before its approval for use against IPF because other types of FILDs had not received significant attention. In recent years, clinicians have increasingly recognized the importance of ILDs (including IPF and other types of fibrosis) due to introduction of the PF-ILD concept and ATS/ERS/JRS/ALAT PPF guidelines. A meticulous discussion and assessment of the efficacy of antifibrotic agents in FILD management are needed, which highlights the importance of exploring alternative treatment options for individuals facing challenges in managing their condition. ATS/ERS/JRS/ALAT PPF guidelines recommend that further research and evidence are needed to fully understand the efficacy and safety of pirfenidone across various subtypes of PPF.

We systematically compiled data on pirfenidone use in diverse FILDs, revealing its widespread utilization across most of these conditions. Pirfenidone has emerged as a valuable therapeutic option for patients suffering from different FILDs. These conditions encompass a range of disorders characterized by progressive scarring and inflammation in the lungs: IPF, CTD-ILD, and chronic hypersensitivity pneumonitis. Data demonstrate that pirfenidone is being used across these diverse conditions because it can slow down disease progression and improve quality of life. Pirfenidone reduces excessive deposition of collagen, inhibits inflammatory processes, and suppresses fibroblast proliferation, thereby preventing further damage to the lungs. Pirfenidone is safe and well-tolerated by patients with non-IPF FILDs, further supporting its use beyond IPF. This consistency in safety profile suggests that pirfenidone could be a viable therapeutic option for a broader range of patients suffering from various forms of FILD.

Despite encouraging findings, the level of evidence regarding the efficacy of pirfenidone is limited. Clinical trials assessing its utility in specific forms of FILD (e.g., RA-ILD, IPAF) have yielded valuable insights, but have been relatively small-scale or lacking long-term follow-up data. The COVID-19 pandemic significantly impeded progress in several randomized controlled trials conducted within the past 5 years, leading to delays in participant enrollment or follow-up. Further research is needed to establish deeper understanding of the efficacy and safety profile of pirfenidone across indicates the company types of FILD (Table 2).

**TABLE 2:** Investigational drugs undergoing clinical trials currently.

Registration number	Disease	Phase	Country in which trial is being held	Registration date
NCT05505409	Connective-tissue diseases; interstitial lung disease	4	China	2022-08-16
NCT03939520	Progressive idiopathic pulmonary fibrosis	4	France	2019-05-03
ChiCTR2100050555	Coal workers’ pneumoconiosis	4	China	2021-08-28
ChiCTR2100044973	Radiation-induced fibrosis	4	China	2021-04-03
ChiCTR2100044854	Idiopathic inflammatory myopathy	4	China	2021-03-30
ChiCTR2100043032	Radiation-induced lung injury	4	China	2021-02-04
ChiCTR2100042720	Interstitial lung disease	4	China	2021-01-26
ChiCTR2000037602	Interstitial lung disease	4	China	2020-08-29
ChiCTR1900021654	Urethral stricture	4	China	2019-03-03
EUCTR2016-003827-45-GB	Idiopathic pulmonary fibrosis	4	Greece; Spain; United Kingdom	2018-02-01
CTRI/2021/09/036442	Post-COVID-19 lung disease	3	India	2021-09-13
EUCTR2020-005306-25-IT	Acute respiratory distress syndrome	3	Italy	2021-06-07
EUCTR2019-004326-19-FR	Idiopathic pulmonary fibrosis	3	France	2019-11-13
JPRN-UMIN000029411	Non-small-cell lung cancer combined with idiopathic pulmonary fibrosis	3	Japan	2017-10-15
NCT05801133	Lung cancer	2	China	2023-03-24
NCT05704166	Acute Lung Injury; prevention	2	China	2022-12-21
NCT05118256	Silicosis; progressive massive fibrosis; complicated silicosis	2	Spain	2021-10-01
ChiCTR2200064594	Radiation-induced lung injury	2	China	2022-10-12
EUCTR2020-002518-42-ES	Pulmonary fibrosis induced by SARS-COV-2 infection (post-COVID-19 pulmonary sequelae)	2	Spain	2020-10-28
EUCTR2018-001781-41-NL	Asbestosis	2	Netherlands	2018-11-01
NCT05280873	Pneumonitis; malignant tumor	1	China	2021-10-11

Data were updated to February 20, 2024.

## 8 Conclusion

The potential of pirfenidone in the treatment of various fibrotic interstitial lung diseases is high, but evidence is insufficient. Nevertheless, pirfenidone has demonstrated tolerability. Further investigations into the efficacy of pirfenidone across different subtypes within this disease category are eagerly awaited due to the existing dearth of evidence. Our comprehensive review provides a valuable reference for future clinical investigations into the efficacy and safety associated with pirfenidone use.

## Author contributions

ZG: Conceptualization, Data curation, Funding acquisition, Project administration, Resources, Supervision, Validation, Writing – review & editing. LJ: Formal analysis, Writing – original draft, Funding acquisition. WJ: Formal analysis, Methodology, Funding acquisition, Writing – review & editing. MH: Data curation, Formal analysis, Investigation, Methodology, Visualization, Writing – original draft. QL: Formal analysis, Funding acquisition, Resources, Writing – review & editing. ZJ: Formal analysis, Resources, Funding acquisition, Writing – review & editing.
