# Correction: Use of pirfenidone in fibrotic interstitial lung diseases and beyond: a review

**DOI:** 10.3389/fmed.2026.2019848

**Published:** 2026-10-23

**Authors:** Mingfeng Han, Qijia Liu, Zhe Ji, Lili Jin, Wenyu Jin, Zhonggao Gao

**Affiliations:** 1 School of Pharmacy, Yanbian University, Yanji, Jilin, China; 2 Ruibo International Business School, Beijing, China; 3 School of Finance, Renmin University of China, Beijing, China; 4 Department of Dermatology, Yanbian University Hospital, Yanji, Jilin, China; 5 State Key Laboratory of Bioactive Substance and Function of Natural Medicines, Department of Pharmaceutics, Institute of Materia Medica, Chinese Academy of Medical Sciences and Peking Union Medical College, Beijing, China

**Keywords:** fibrotic interstitial lung diseases, pirfenidone, CTD-ILD, Post-COVID-19 pulmonary fibrosis, adverse events

The funder Natural Science Foundation of Jilin Province, grant number 232679JC0105106665, to Lili Jin was erroneously omitted.

The original version of this article has been updated.

